# Cytotoxic effects of crotoxin from *Crotalus durissus terrificus* snake in canine mammary tumor cell lines

**DOI:** 10.1590/1678-9199-JVATITD-2023-0062

**Published:** 2024-03-18

**Authors:** Giovana Pedro, Felipe César da Silva Brasileiro, Jamile Mariano Macedo, Andreimar Martins Soares, Gabriel Caporale Mafra, Carlos Eduardo Fonseca Alves, Renée Laufer-Amorim

**Affiliations:** 1 School of Veterinary Medicine and Animal Science, São Paulo State University (UNESP), Botucatu, SP, Brazil.; 2 Laboratory of Biotechnology and Education Applied to One Health (LABIOPROT), Oswaldo Cruz Foundation, Fiocruz - Porto Velho, RO, Brazil.; 3 Federal University of Rondônia (UNIR), Porto Velho, RO, Brazil.; 4 São Lucas University Center - São Lucas PVH, Porto Velho, RO, Brazil.; 5 Western Amazon Research and Knowledge Network of Excellence (RED-CONEXAO), Porto Velho, RO, Brazil.; 6 National Institute of Science and Technology of Epidemiology of the Western Amazon (INCT EpiAmO), Porto Velho, RO, Brazil.

**Keywords:** Mammary tumor, Comparative oncology, *Crotalus durissus terrificus* venom, Biological compounds

## Abstract

**Background:**

Mammary gland tumors are the most prevalent neoplasm in intact female dogs, and they are good natural models to study comparative oncology. Most canine mammary malignancies, as in women, are commonly refractory to conventional therapies and demand continuous new therapeutic approaches. *Crotalus durissus terrificus*, also called rattlesnake, has more than 60 different proteins in its venom with multiple pharmaceutical uses, such as antitumor, antiviral, and antimicrobial action. Crotoxin, a potent β-neurotoxin formed by the junction of two subunits, a basic subunit (CB-PLA_2_) and an acidic subunit (crotapotin), has already been reported to have anticancer properties in different types of cancers.

**Methods:**

In this work, we describe the cytotoxic potential of crotoxin and its subunits compared to doxorubicin (drug of choice) in two canine mammary carcinoma cell lines.

**Results:**

Crotoxin, CB-PLA_2_, crotalic venom, and doxorubicin decreased cell viability and the ability to migrate in a dose-dependent manner, and crotapotin did not present an antitumoral effect. For all compounds, the predominant cell death mechanism was apoptosis. In addition, crotoxin did not show toxicity in normal canine mammary gland cells.

**Conclusion:**

Therefore, this work showed that crotoxin and CB-PLA_2_ had cytotoxic activity, migration inhibition, and pro-apoptotic potential in canine mammary gland carcinoma cell lines, making their possible use in cancer research.

## Background

Mammary gland tumors are the most prevalent neoplasm in intact female dogs, but the incidence can vary and be as high as 83% in a study population [1, 2]. Comparatively, in 2022, Brazil’s public health system reported 73,610 new diagnoses of breast cancer in women, 30.1% of all newly diagnosed neoplasias, which is more than three times what ranked second in statistics [3].

Canine cancers occur spontaneously and have a similar clinical presentation and pathophysiology to humans, in addition to sharing the main risk factors and signaling pathways [4, 5], such as the expression of the HER-2 protein and Ki-67, related to tumor aggressiveness. This makes dogs a valuable study model as they are more similar to the natural progression of human cancer than induced animal cancer models [6]. Most canine mammary malignancies are commonly refractory to conventional therapies and demand continuous new therapeutic approaches, as in women. Biological compounds are the source of 25% of newly approved medicines used in cancer treatment in recent decades in natural or re-engineered structures [7, 8].

As a source of multiple biological molecules, snake venom is a tremendous opportunity for new medical uses by improvement with a biotechnological process [9, 10, 11, 12, 13, 14]. *Crotalus durissus terrificus*, a Viperidae snake (Crotalinae subfamily) also called Rattlesnake, and its venom (CdtV) comprises more than 60 different proteins with multiple pharmaceutical uses, such as antitumor, antiviral, and antimicrobial activities [9, 15, 16]. Crotoxin (CTX) is a potent β-neurotoxin formed by the junction of two subunits: one basic (CB-PLA_2_) enzymatic part and another acidic (crotapotin) with structural properties, exhibits nephrotoxic, cardiotoxic, and myotoxic effects [17] and this complex protein represents approximately 50% of dry crude venom [9, 12, 18, 19, 20, 21]. 

In recent decades, CTX has been the most analyzed toxin derived from crude snake venom and used as a multimodal natural agent, with functions such as immunomodulatory, antimicrobial, anticancer, anti-inflammatory, and analgesic compounds [20, 22, 23, 24, 25]. The ability to regulate cytotoxin production in the microenvironment, modulate cell proliferation signaling, promote cell cycle arrest or induction of apoptosis/autophagy, and specifically activate/deactivate cell membrane receptors are the main mechanisms of action in cancer cells [18, 26].

This neurotoxin has been studied *in vitro*, *in vivo*, and more recently in clinical trials, alone or in combination, to develop new human medicines [27, 28, 29]. In canine models, this promisor agent, to the best of our knowledge, has not yet been reported. This study aimed to describe the cytotoxic potential and migration inhibition capacity of crotoxin and its subunits compared to doxorubicin (drug of choice) in two canine mammary carcinoma cell lines. 

## Methods

### Materials and chemicals

The reagents used were: antibiotic antimycotic - solution (Gibco™, Thermo Fisher Scientific); APC conjugated with Annexin V (Invitrogen™, Thermo Fisher Scientific); dimethylsulfoxide (Dinâmica, Sigma-Aldrich®); Dulbecco’s modified Eagle’s medium Ham F-12 (Merk, Sigma-Aldrich®); Dulbecco’s phosphate buffered saline (LGC Biotecnologia®); fetal bovine serum (Nova Biotecnologia®); gentamicin (Gibco™, Thermo Fisher Scientific); Hoechst (Invitrogen™, Thermo Fisher Scientific); propidium iodide (Invitrogen™, Thermo Fisher Scientific); Trypsin-EDTA solution (Gibco™, Thermo Fisher Scientific); 3-(4,5-dimethyl-2-thiazolyl)-2,5-diphenyl-2-H-tetrazolium bromide - MTT (Invitrogen™, Thermo Fisher Scientific).

### Venom and fraction preparations

*Crotalus durissus terrificus* venom (CdtV) was obtained from the Serpentarium of the University of São Paulo, Ribeirão Preto School of Medicine, Ribeirão Preto, São Paulo, Brazil. The licenses related to access to Brazilian genetic resources for scientific purposes are the authorizations: CGEN/CNPq 010627/2011-1; IBAMA 27131-2 and CEBio UNIR-FIOCRUZ-RO (register CGEN A4D12CB and IBAMA/SISBIO 64385-1).

A total of 250 mg of the dry venom was dispersed in 2.5 mL of 0.05 M ammonium formate buffer (NH_4_HCO_2_) pH 3.5 and centrifuged at 755 *xg* for 10 min at room temperature (25 °C). The clear supernatant was then applied to a Sephadex G-75 column equilibrated with the same buffer; fractions of 0.1 mL/tube were collected and monitored at Abs 280 nm using an automatic fraction collector. Purification of crotoxin and subunits [crotoxin A (crotapotin) and crotoxin B (CB or Asp 49-PLA2)] was performed as described elsewhere with minimal changes [30, 31, 32, 33, 34, 35]. The elution was monitored at an absorbance of 280 nm, manually collected, lyophilized, and stored at -20 °C.

### Cell lines and cell culture

Mammary tumor samples were collected at the Veterinary Hospital of the School of Veterinary Medicine and Animal Science of UNESP, Botucatu. The fragments were placed in a culture medium (DMEM), added with 1% gentamicin and 0.5% antibiotic-antimycotic solution, and supplemented with 10% fetal bovine serum. For *in vitro* expansion, the collected neoplasm fragments measuring approximately one cm² were dissociated with the type IV collagenase enzyme for four hours. Plating was done at a concentration of 10^4^ cells/mL in culture bottles with a volume equal to 25 mL. After achieving 80% confluence in the bottle, the cells were trypsinized and placed in new 75 cm² bottles with a filter. The cell lines used, UNESP-CM1 and UNESP-CM9, were previously established and characterized by Lainetti [36]. UNESP-CM1 comes from a 12-year-old poodle and was classified as a solid carcinoma, grade II with tubular formation and HER2 overexpressing. UNESP-CM9 comes from a 12-year-old mixed breed and was classified as a tubulopapillary, grade II with tubular formation and HER2 overexpressing. The cells used were in their logarithmic growth phase in all experiments.

### *In vitro* cytotoxicity assay 

The colorimetric MTT assay was used to determine the cytotoxic effect of crotoxin, PLA_2_ (CB-PLA_2_), crotapotin, CdtV, and doxorubicin. For this, 1×10^4^ cells/well of UNESP-CM1 and UNESP-CM9 were seeded in a 96-well plate at different concentrations of the compounds. After 24 hours of seeding in DMEM with 10% FBS, the media was replaced by DMEM without BFS plus the different concentrations of the tested compounds under the same conditions and for the same period (24 hours). For crotoxin, the doses tested were: 120, 240, 360, and 480 μg/mL. For CB-PLA_2_: 72.5, 145, 217.5, 290 μg/mL. For Crotapotin: 48, 96, 44, 192 μg/mL. For *Crotalus durissus terrificus* venom: 60, 120, 240, 480 μg/mL. For doxorubicin: 1.5, 3, 6, 12; 9, 18, 36, 72 μg/mL.

The MTT assay (Invitrogen™, Thermo Fisher Scientific, USA) was performed according to the manufacturer’s instructions, and Spectro colorimetric analysis was performed in a microplate reader (570 nm range). All the compounds were tested in four concentrations made in a serial dilution, guided by control groups, and executed in triplicate. GraphPad Prism 8.0.1 software was used to normalize the Spectro colorimetric data, plot a nonlinear regression, and determine the IC_50_ by the dose-response curve. The treated groups and control were compared by individual t-tests considering p < 0.05 to be significant.

### Cellular migration

The inhibition potential for cellular migration was tested by a wound healing test after treatment with the IC_50_ doses of crotoxin, PLA_2_ (CB-PLA_2_), crotapotin, and doxorubicin. For this, the cells were seeded in a 6-well plate (4.5×10^5^ cells/well) until they reached 80% confluence. After that, a 100 µL pipette tip was used to trace a linear wound. The plates were double-washed with DPBS (500 µL) and 5 min agitation to remove the detached cells.

For reference, each well was photographed at the time the wound was made and after 24 hours. During this time, the cells were incubated in fresh DMEM (without FBS), and the compound testing dosage was added. Wound healing was measured in five different regions using the GIMP 2.10.14 program. The mean distance for each cell line was calculated as the mean and standard deviation. The treated groups and control were compared by individual T-tests considering p < 0,05.

### Apoptosis analysis

The cell death analysis of UNESP-CM1 and UNESP-CM9 was performed after 24 hours of treatment with the IC_50_ dose of all the tested compounds. The samples were suspended in a medium containing calcium (buffer solution) for analysis of apoptosis. To this end, 5 µL of APC-conjugated annexin V (Becton Dickinson and Company), 5 µL (1.5 µM final concentration) of propidium iodide (Becton Dickinson and Company), and 5 µL (7 µM final concentration) of Hoechst (Sigma, USA) were added to the cell suspensions.

All samples were incubated in the dark for 15 min at room temperature, and flow cytometry assessment was performed with a final concentration of 1×10^6^ cells/mL in Fortessa LSR equipment (Becton Dickinson, Mountain View, CA, USA). The filter configurations for the PMTs measuring fluorescence emission of the applied fluorochromes were 694/50 nm (IP), 660/20 nm (Annexin-APC), and 450/50 nm (Hoechst 33342). The acquisition rate was 800 events per second, and at least 1×10^4^ cells were analyzed per sample.

Data were generated in a contour plot graph including axis < 0t (biexponential), making all events visible and properly compensated through BD FACSDiva TM software v6.1 (Becton Dickinson).

### Cytotoxicity analysis of crotoxin in normal mammary gland cells

Normal canine mammary glands were collected for histopathological examination and cell culture, and the fragment was processed by enzymatic dissociation with collagenase type IV for 4 hours. The resultant material was filtered and centrifuged in a controlled environment, with subsequent seeding in a 25 cm^2^ cell culture flask with DMEM until 80% confluence was obtained. Cytotoxicity analysis was tested for crotoxin in the same manner as for the tumor cell lines.

### Data analysis

The IC_50_ values of all the tested compounds were obtained by plotting the spectrum colorimetric data in a dose-response curve after the application of data normalization and a nonlinear regression method in GraphPad Prism 8.0.1 software. The values presented are the mean and standard deviation of triplicate tests, and statistical significance (p < 0,05) was obtained by a comparison of each tested group with the control (vehicle) by independent t-tests and ANOVA.

For the cell migration analysis, the mean and standard deviation were calculated. The wound healing (difference) was taken by the formula D0-D1, of which D0 was the first measure, and D1 was the measure compared to the initial moment. The treated groups and control were compared using the Mann‒Whitney test, considering p < 0.05 as an indicator of statistical significance, and this was done in GraphPad Prism 8.0.1 software.

## Results

### Venom and fraction preparations

**Figure 1.**
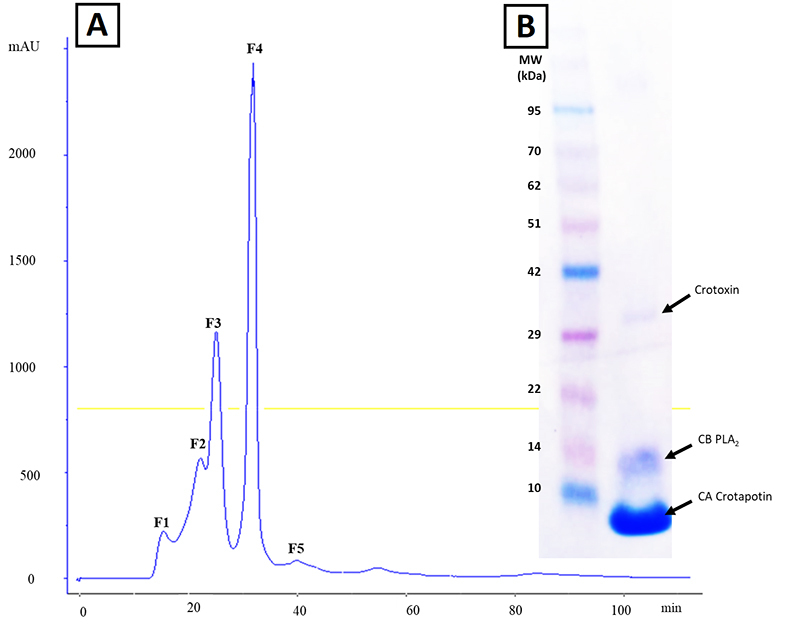
Chromatographic profile of the *Crotalus durissus terrificus* venom pool, where four fractions were observed, the third peak was suggestive of crotoxin. On the X axis, the time measured in minutes, on the Y axis on the right, the concentration of eluent B and on the Y axis on the left, the optical density expressed in AU (280 nm). On the left, the SDS-PAGE gel of the third peak, with the presence of two distinct bands, suggestive of crotapotin (8.9 kDa) and PLA_2_ (14.3 kDa).

Figure 1 shows the chromatographic profile of the venom of *Crotalus d. terrificus* (Figure 1A ), with five fractions, F4 being the crotoxin, as shown in the electrophoresis gel (Figure 1B).

### Cellular Viability after toxin exposure

**Figure 2.**
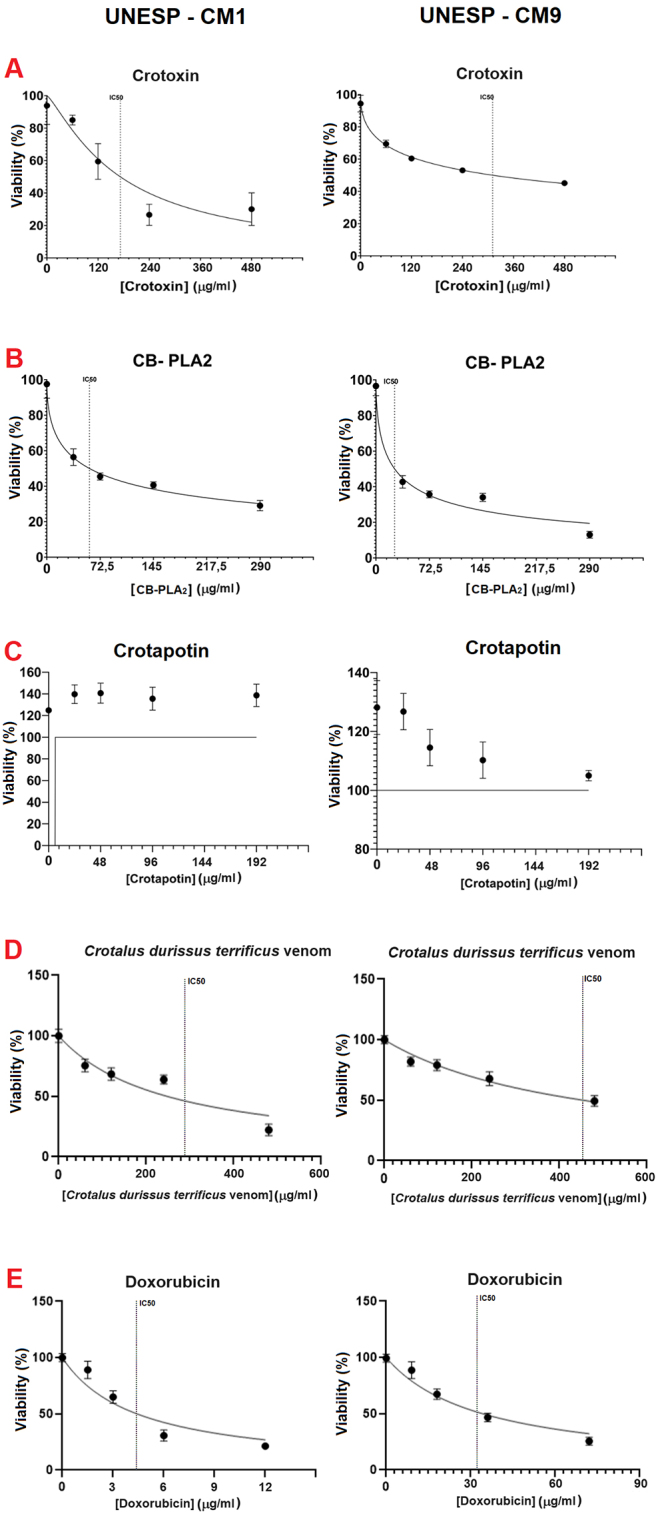
Cytotoxicity assay (MTT - 24 h) on the canine mammary carcinoma cell lines UNESP-CM1 (left) and UNESP-CM9 (right). ( **A**) Note the decrease in cell viability as there is an increase in crotoxin (CTX) concentrations for both cell lines. ( **B**) Note the decrease in cell viability as there is an increase in CB-PLA_2_ concentrations for both cell lines. ( **C**) Note the absence of a toxic effect of crotapotin on both cell lines. ( **D**) Note the decrease in cell viability as there is an increase in the concentrations of *Crotalus durissus terrificus* venom in both cell lines. ( **E**) Note the decrease in cell viability with doxorubicin in a dose-dependent manner in both canine mammary carcinoma cell lines.

For both canine mammary carcinoma cell lines (UNESP-CM1 and UNESP-CM9), there was a decline in cellular metabolic activity by the increase in CTX, CB-PLA_2_, crotalic venom, and doxorubicin in a dose-dependent manner (Table 1 and Figure 2). For the acid component of crotoxin (crotapotin), we did not find an antitumoral effect.

**Table 1.** IC_50_ of the drugs tested in UNESP-CM1 and UNESP-CM9, two canine mammary carcinoma cell lines.

Drug	UNESP-CM1		UNESP-CM9	
	IC_50_	R^2^	IC_50_	R^2^
CTX	172.08 μg/mL	0.8327	310.8 μg/mL	0.9571
CB-PLA_2_	57.98 μg/mL	0.9570	25.43 μg/mL	0.9353
CdtV	285 µg/mL	0.8338	456.2 µg/mL	0.8829
Doxorubicin	4.29 μg/mL	0.8711	33.23 μg/mL	0.9196

### Cellular migration

**Figure 3.**
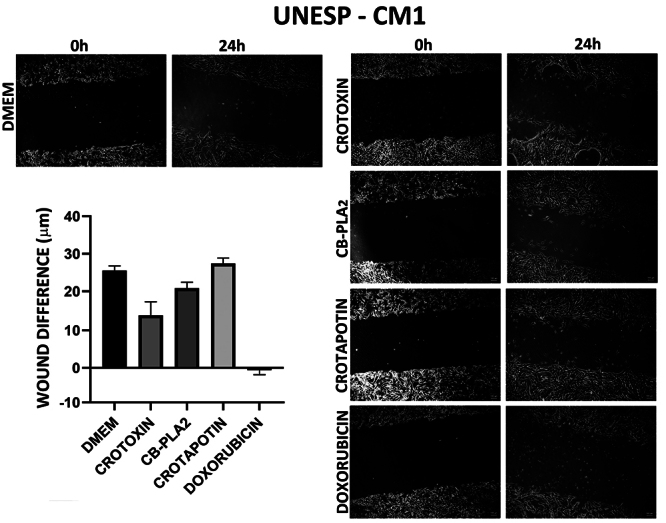
Wound healing test in the tested and control groups at 0 and 24 h in the UNESP-CM1 canine mammary gland carcinoma cell line.

**Figure 4.**
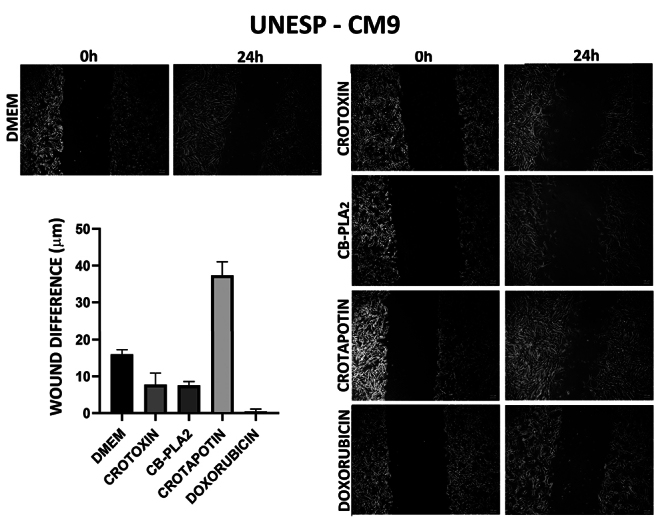
Wound healing test in the tested and control groups at 0 and 24 h in the UNESP-CM9 canine mammary gland carcinoma cell line.

For UNESP-CM1 and UNESP-CM9, when comparing the mean of closure of the wound difference between 0 h and 24 h, Crotoxin, CB-PLA_2_, and doxorubicin showed an effect on delaying wound healing, but with no significant difference ( Figures 3 and 4 and Table 2). Crotapotin did not inhibit cell migration.

**Table 2.** Wound measurement and healing percentage in 24 h intervals.

Cell	Group	0 h	24 h	Wound (μm)	Healing (%)	p Value
		*x̄*	*x̄*			
CM1	DMEM	167.3	141.8	25.50 ± 2.49	15.24%	
	Crotoxin	161.9	147.9	13.98 ± 4.48	8.67%	0.0006
	CB PLA_2_	148.8	128.0	20.80 ± 1.45	13.98%	0.0041
	Crotapotin	137.6	110.2	27.40 ± 1.54	19.91%	0.0991
	Doxorubicin	165.4	166.0	-0.60 ± 1.98	-0.36%	< 0.0001
CM9	DMEM	120.9	104.9	15.98 ± 1.50	13.20%	
	Crotoxin	138.8	131.0	7.75 ± 1.98	5.58%	0.0029
	CB PLA_2_	164.6	157.1	7.50 ± 3.17	4.56%	< 0.0001
	Crotapotin	132.9	95.5	37.35 ± 3.15	28.13%	< 0.0001
	Doxorubicin	147.7	147.3	0.40 ± 1.34	0.26%	< 0.0001

### Mechanism of cell death

**Figure 5.**
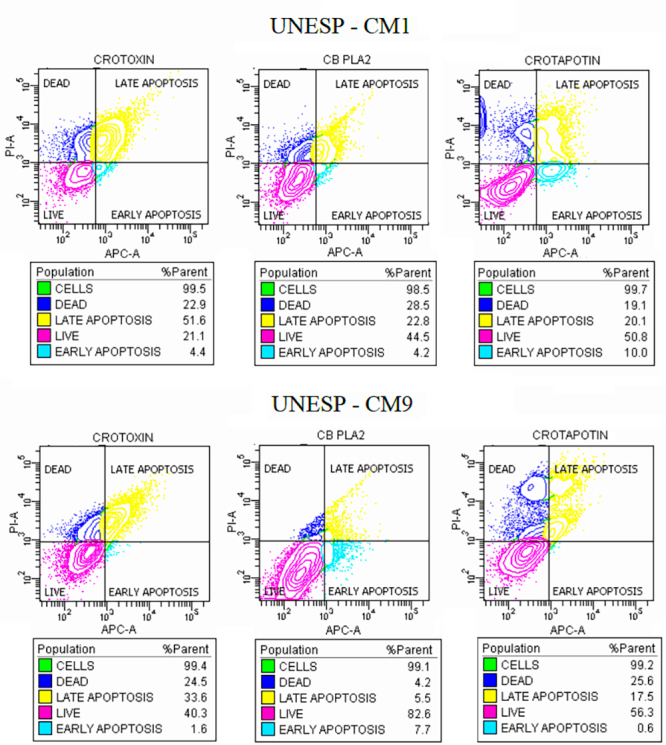
Apoptosis Assay (Annexin V x Propidium Iodide): Note that crotoxin and crotapotin induced apoptosis of the UNESP-CM1 cell line. CB-PLA_2_ preferentially induced death by necrosis. Note that crotoxin and CB-PLA_2_ induced apoptosis of the UNESP-CM9 cell line. Crotapotin preferentially induced death by necrosis.

Apoptosis was the main cell death mechanism for crotoxin and doxorubicin. The compound with the highest apoptotic potential was crotoxin. Late apoptosis was more frequently detected than premature apoptosis in all samples except CB-PLA_2_ for UNESP-CM9 (Figure 5).

### Normal breast cell cytotoxicity analysis

**Figure 6.**
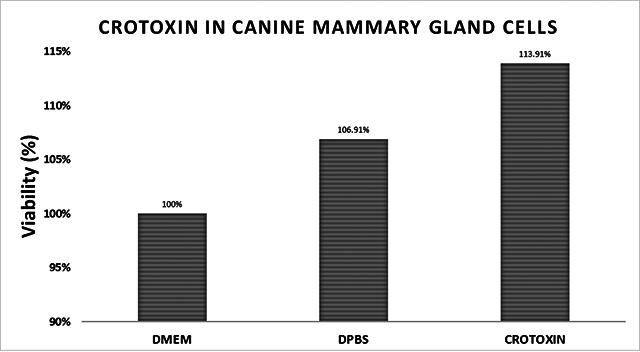
Crotoxin effect on normal canine mammary cells at a dose of 312 µg/mL.

For normal canine mammary cells, 312 µg/mL crotoxin did not show a cytotoxic effect when compared to the same cells treated with DPBS (vehicle) or DMEM (Figure 6). When a similar dose was used in canine mammary tumor cells (UNESP CM-1 and UNESP CM-9), cytotoxic effects and more than 50% mortality were observed in these cells.

## Discussion

*Crotalus durissus terrificus* venom has been tested *in vitro* as an antitumoral drug in breast cancer cell lines [18, 37] but not yet in canine mammary carcinoma cells. Considering that canine mammary tumors are a good natural model for comparative oncology studies, we tested *Crotalus durissus terrificus* venom in two canine mammary carcinoma cell lines, with results similar to human breast cancer cell lines. Other human cancer cell lines have been tested for the antitumoral effects of crotoxin, such as lung, pancreas, cervix, colon, kidney, ovary, esophagus, brain, melanoma, glioma, and squamous cell carcinoma [38, 39, 40, 41, 42].

Crotoxin is the main toxin present in rattlesnake venom and is composed of two subunits: an acidic subunit (crotapotin) and one basic subunit (CB-PLA_2_). In our study, the basic subunit was responsible for the cytotoxic activity of crotoxin, showing an IC_50_ of 25.43 and 57.98 μg/mL. In contrast, the acidic subunit did not show such effects at a 192 μg/mL dose. This result is in accordance with the study carried out by Corin [43] with MEL cells from murine erythroleukemia, where crotoxin showed an IC_50_ of 0.8-1.0 µg/mL for these cells, without showing toxicity of crotapotin at 20 µg/mL. In addition, the IC_50_ of isolated CB-PLA_2_ is lower than that of crotoxin, which allowed us to perceive that this secluded molecule may be more adequate because it has a minor molecular weight, and this fact directly influences the use of drug transporters and penetration into cell membranes, optimizing their effectiveness as a therapeutic agent.

The effects of crotoxin in normal canine breast cells at a 312 µg/mL dose showed no toxicity for these cells, as proven by other authors. Almeida [18] showed that crotoxin is a secure compound for non-tumor cells (HFF-1) at 100 μg/mL doses. Rudd [44] demonstrated low toxicity of CTX in normal cells, indicating selectivity for tumor cells. In this experiment with normal canine mammary cells, we only use crotoxin, and this is justified since the subunits are mixed and present in the complete protein, adding to the already known toxic effects of doxorubicin in normal cells.

Muller [37] also showed this specific cytotoxicity using mouse fibroblasts (3T3) and human keratinocytes (HaCaT). At a 30 μg/mL dose, crotoxin was not toxic to these cells, which are strongly affected by antineoplastic chemotherapies [45]. However, it showed a significant drop in viability in pancreatic cancer (PSN-1 and PANC-1), esophageal cancer (Kyse 30), cervical cancer (HeLa), and glioma (GAMG, HCB 151 and U373) cell lines. The most resistant cell lines were cervical (SiHa) and esophageal (KYSE270) carcinoma. Although crotoxin is toxic to all cell lines, it presents a very heterogeneous response, even in the same type of tumor, which corroborates our study where the IC_50_ values for two different canine mammary tumor cell lines were 172.08 µg/mL and 310.80 µg/mL for UNESP-CM1 and UNESP-CM9, respectively.

In our study, crotoxin inhibited cell migration, presenting a lower healing percentage than the control group for CM1 8.67% and CM9 5.58%, compared to 13.20% and 15.24% in the control group for the respective cell lines. In the same test, Da Rocha [42] also demonstrated inhibition of migration in oral squamous carcinoma cells, making it a promising compound in the oncology field since migration is one of the bases of the mechanism of metastasis.

The crotoxin in this experiment showed that apoptosis was the most prevalent cell death mechanism. We observed 56% apoptosis versus 22.9% necrosis for UNESP-CM1 and 35.2% apoptosis versus 24.5% necrosis for UNESP-CM9 cells. A similar method has already been identified in other models of cancer cells by other authors. Han et al. (2014) in human lung squamous carcinoma (SK-MES-1) and Ye [46] in human lung adenocarcinoma (A549) demonstrated apoptosis by increasing p38 MAPK and caspase 3. Another mechanism of action of crotoxin was the reduction of angiogenesis and tumor growth in a xenograft model [46]. He [ 40] showed that CTX induces caspase 3 in human esophageal carcinoma (Eca-109). Almeida [18] demonstrated in ER+ breast cancer cells that CTX induces apoptosis mediated by caspase-8. Da Rocha [42] showed reduced cell viability by increasing DNA damage, in addition to reducing the expression of MMP9, MM2, and COL1A1 (proteins related to invasion and metastasis).

Similar to other medicines earned by snake venom sources, the multimodal benefits promised exerted by crotoxin in canine oncologic patient treatment bring new possibilities. The results of a phase I clinical trial of CTX performed in patients with untreatable tumors were published in 2002. Limited neurological toxic effects were observed, but all of these effects disappeared completely during the study. Of the 23 patients, two had a tumor mass reduction greater than 50%, one with a measurable tumor in the vaginal region, had a reduced tumor mass allowing for surgical removal, and one patient had a complete response [27].

## Conclusion

We confirmed that crotoxin can exert cytotoxic effects in canine mammary carcinoma cells (UNESP-CM1 and UNESP-CM9) in addition to being noncytotoxic to normal canine breast cells. This use of crotoxin in canine mammary carcinoma cell lines reveals the success of this toxin in promoting apoptosis, opening a promising opportunity for further studies *in vivo*, using the dog as a natural model for mammary carcinoma clinical trials.

### Abbreviations

CTX: crotoxin; CB-PLA_2_: PLA_2_ of crotoxin; CdtV: *Crotalus durissus terrificus* venom; DMSO: dimethylsulfoxide; DMEM: Dulbecco’s modified Eagle’s medium; DPBS: Dulbecco’s phosphate buffered saline; FBS: fetal bovine serum.

## Data Availability

The datasets generated during and/or analyzed during the current study are available from the corresponding author upon reasonable request.
